# GSH-responsive poly-resveratrol based nanoparticles for effective drug delivery and reversing multidrug resistance

**DOI:** 10.1080/10717544.2021.2023700

**Published:** 2022-01-08

**Authors:** Yang Liping, He Jian, Tao Zhenchao, Zhou Yan, Yang Jing, Zhang Yangyang, Gao Jing, Qian Liting

**Affiliations:** Department of Radiotherapy Oncology, the First Affiliated Hospital of USTC, Division of Life Sciences and Medicine, University of Science and Technology of China, Hefei, China

**Keywords:** Polymer-based nanomedicine, resveratrol, GSH-sensitive drug, multidrug resistance

## Abstract

Cancer poses a serious threat to human health and is the most common cause of human death. Polymer-based nanomedicines are presently used to enhance the treatment effectiveness and decrease the systemic toxicity of chemotherapeutic agents. However, the disadvantage of currently polymeric carriers is without therapy procedure. Herein, for the first time, glutathione (GSH)-responsive polymer (PRES) with anti-cancer effect was synthesized following the condensation–polymerization method using resveratrol (RES) and 3,3′-dithiodipropionic acid. PRES can not only suppress the tumor cells growth but can also self-assemble into nanoparticles (∼93 nm) for delivering antitumor drugs, such as paclitaxel (PTX@PRES NPs). The system could achieve high drug loading (∼7%) and overcome multidrug resistance (MDR). The results from the *in vitro* studies revealed that the NPs formed of PRES were stable in the systemic circulation, while could be efficiently degraded in tumor cells high GSH environment. Results from cytotoxicity tests confirmed that PTX@PRES NPs could effectively suppress the growth of cancer cells (A549) and drug-resistant cells (A549/PTX). The NPs could also be used to significantly increase the therapeutic efficacy of the drugs in A549/PTX tumor-bearing mice. *In vivo* investigations also demonstrated that the PRES-based NPs exhibited tumor inhibition effects. In summary, we report that the GSH-responsive polymer synthesized by us exhibited multiple interesting functions and could be used for effective drug delivery. The polymer exhibited good therapeutic effects and could be used to overcome MDR. Thus, the synthesized system can be used to develop a new strategy for treating cancer.

## 1. Introduction

Cancer severely threatens human life in all the countries of the world. In 2020, over 19.3 million new cases of cancer and nearly 10.0 million cancer-related deaths were reported across the globe (Sung et al., 2021). Cancer is widely treated following the process of chemotherapy (Yang et al., 2018). Attention is being paid to the development of nanomedicines to enhance the therapeutic effect and minimize the side effects of chemotherapeutics. It is believed that the use of nanomedicines can result in increased drug efficacy (Au et al., 2020). The use of nanocarrier-based delivery systems can increase the solubility of hydrophobic drugs (Tan et al., 2019), enhance the bioavailability of drugs (Rosenblum et al., 2018), improve the accumulation of drugs at tumor tissues by improving the permeability and retention (EPR) effects (Tee et al., 2019), and result in decreased side effects (Raj et al., 2021). Developing suitable drug carriers is the elementary step to prepare the nano-drug delivery system. Various biomaterials, such as liposomes, polymers, exosomes, cell membranes, and peptides are being used to construct nano-drug delivery systems (Hu et al., 2016). Polymers have been widely used in the field of anticancer drug delivery (Pottanam Chali & Ravoo, 2020). The complex synthetic process, absence of biological activity, and a high degree of toxicity significantly limited the practical applications of the developed polymeric drug carriers in clinical settings (Shi et al., 2015; Ma et al., 2020) Hence, it is important to develop polymers that can be easily synthesized and used during treatment (Zheng et al., 2019; Ou et al., 2020). To date, few studies have been conducted on such polymer-based-drug carriers.

3,4′,5-trihydroxy-*trans*-stilbene, also known as Resveratrol (RES), is a natural polyphenolic phytoalexin found in 185 plant species. It is found in red wine, soybeans, peanuts, berries, etc. (Jhaveri et al., 2018). It exhibits a wide range of biological (such as anticancer, anti-carcinogenic, cardio-protective, neuroprotective, immunomodulatory, anti-inflammatory, and anti-oxidant) activities (Santos et al., 2019). RES is a potential anticancer molecule that suppresses the proliferation of various cancerous cells, such as breast, stomach, prostate, skin, colon, lung, and liver cells (Huminiecki & Horbańczuk, 2018). It has been reported that the use of RES can result in the recovery of the lost sensitivity of cells toward drugs, such as paclitaxel (PTX), doxorubicin (DOX), and methotrexate (MTX) (Alamolhodaei et al., 2017). Thus, RES can be used to reverse the case of multidrug resistance (MDR) to some extent. Poor water solubility, chemical instability (photosensitivity and auto-oxidation), ease of clearance, and low tumor-targeting ability have significantly limited the application of RES (Jangid et al., 2020).

Various nanosized systems were developed to achieve the delivery of RES to address these problems (Jhaveri et al., 2018; Jangid et al., 2020). These delivery systems developed for RES can be classified into two types. One type of system is used to encapsulate RES to form nanocarriers, such as polymeric micelles, protein-based nanoparticles, liposomes, and inorganic nanoparticles (Jhaveri et al., 2018; Shen et al., 2018; Singh et al., 2018; Santos et al., 2019; Zhao et al., 2020). Jangid et al. prepared a novel amphiphilic polymer by functionalizing Pluronic F68 with lipid (stearic acid) and polysaccharide (inulin) that could function as a drug carrier (Jangid et al., 2020). RES could be loaded into the developed carrier and used to treat colon cancer. The process resulted in an increase in the blood circulation time and enhanced *in vitro* antitumor effect. As RES bears multiple active hydroxyl groups, another type of delivery system is developed following the process of covalent modification of RES. The process of covalent modification results in a change in the chemical and physical properties of RES. RES could be conjugated with polyethylene glycol (PEG) to increase the solubility, extent of blood circulation, and antitumor effects (Wang et al., 2016). These strategies could be used to significantly increase the blood circulation time and bioavailability of RES. However, the methods are characterized by low drug loading and uncontrolled drug release, resulting in unsatisfactory therapeutic effects.

**Scheme 1.**
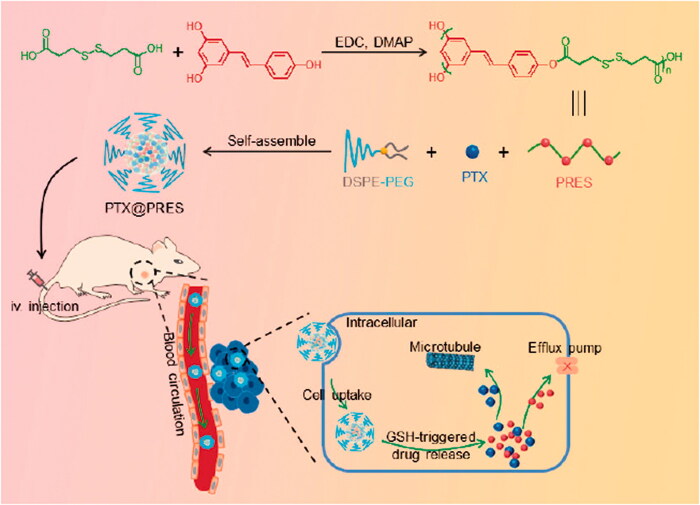
Schematic representation of the process of PRES preparation, self-assembly of GSH-responsive PTX@PRES NPs, mode of application of the fabricated NPs in the field of cancer therapy and, mechanism of reversing MDR.

To the best of our knowledge, RES or RES-based materials have not been developed for use as drug carrier materials to date. RES or RES-based materials can be potentially used to develop drug delivery systems and for treating diseases as these exhibit good biological activities. RES cannot be directly used for drug delivery, but it can be polymerized *via* the three hydroxyl groups with a suitable linker to form polymers that can be used for the development of drug delivery systems. The RES-based polymers can be readily assembled into nanocarriers. As a proof of concept, herein, we synthesized a redox-responsive polymer from RES (PRES) following a simple condensation–polymerization reaction involving RES and 3,3′-dithiodipropionic acid (DTPA). The disulfide bond in PRES was stable in blood but could be efficiently degraded by intracellular reduction agents, such as glutathione (GSH). PRES could self-assemble into nanoparticles and control drug release. It could also be used as a nanocarrier for delivering chemotherapeutic agents. We hypothesized that the nano-platform formed using PRES may also enhance the antitumor effect of drugs and help overcome MDR. To confirm this hypothesis, we chose the widely used anticancer drug PTX and loaded it into the NPs fabricated using PRES (PTX@PRES NPs). The prepared PTX@PRES NPs could be used to (Scheme 1) improve the biocompatibility of RES, achieve high drug loading, and realize GSH-responsive drug release. The released RES could improve the sensitivity of the drug-resistant cancer cells toward PTX.

## 2. Materials and methods

### 2.1. Materials

PTX, RES, DTPA, 1-(3-dimethylaminopropyl)-3-ethylcarbodiimide hydrochloride (EDC), N,N’-dimethylformamide (DMF), dichloromethane (DCM), dimethyl sulfoxide (DMSO), and 4-dimethylaminopyridine (DMAP) were obtained from Aladdin Reagents (Shanghai, China). 1,2-distearoyl-sn-glycero-3-phosphoethanolamine-N-[methoxy(polyethylene glycol)-3000] (DSPE-PEG_3k_) was purchased from Xi’an Ruixi Biological Technology Co., Ltd. (Xi’an, China).

### 2.2. Instruments

^1^H nuclear magnetic resonance (^1^H NMR) spectra were recorded on a Varian U500 (300 MHz) spectrometer. The PRES was detected by a matrix-assisted laser desorption ionization-mass spectrometry (MALDI-MS, UltrafeXtreme, Bruker Daltonics, USA). Particle size and polydispersity (PDI) were determined using the dynamic light scattering (DLS, ZetaPlus, USA) technique. The morphology of the nanoparticles was observed using the transmission electron microscopy (TEM, JEM, Japan) technique. The high-performance liquid chromatography (HPLC) technique was used to analyze RES. The mobile phase consisted of a mixture of 0.5% (v/v) acetic acid in methanol and water (1:1, v/v). A flow rate of 1 mL/min was maintained. The UV–vis detection wavelength was 303 nm. The mobile phase used during the process of PTX analysis using the HPLC technique consisted of methanol/H_2_O (6.5:3.5, v/v). The flow rate was maintained at 1 mL/min, and the detection wavelength was 227 nm.

### 2.3. PRES synthesis

PRES was synthesized by conducting an esterification reaction between DTPA and RES. RES (228.0 mg, 1.0 mmol), DTPA (182.2 mg, 1.0 mmol), EDC (401.1 mg, 2.1 mmol), and DMAP (256.2 mg, 2.1 mmol) was dissolved in 100 mL of DCM, and the solution was stirred at room temperature under an atmosphere of nitrogen. After 72 h, the reaction mixture was concentrated to 10 mL, and 40 mL of cold ethyl acetate was added to it. The solution was stored at 4 °C overnight. The precipitate formed was collected following the process of centrifugation. Following this, the collected precipitate was washed thrice with ethyl acetate. Subsequently, the product was dissolved in DMSO, which was then placed into a dialysis bag [molecular weight cutoff (MWCO): 3500 Da]. The solution was dialyzed against DMSO over a period of 48 h. This was followed by dialysis against distilled water (time: 24 h). Finally, the produced PRES was obtained following the freeze–drying cycle conducted under vacuum.

The structure and average molecular weights of PRES were determined using the ^1^H NMR and MALDI-MS.

### 2.4. Redox-responsive behavior of PRES

The redox sensitivity of PRES was studied using the GPC and HPLC techniques. PRES (1.0 mg) was dissolved in a solvent system consisting of DMF and water (DMF: water = 8:1). Subsequently, GSH was added into the mixture and the final concentration was maintained at 10.0 mM. A fraction of the solution (100 μL) was withdrawn from the system and analyzed using the HPLC and GPC techniques following a 6-h long incubation period.

### 2.5. Preparation and characterization of the NPs

The classical nanoprecipitation method was used to prepare the PRES NPs and PTX loaded NPs. For the preparation of the PRES NPs, 100.0 μL of the PRES solution (20 mg/mL in DMSO) and 100 μL of the DSPE-PEG_3k_ solution (20 mg/mL in DMSO) were mixed with each other under conditions of ultrasonication. Following this, the mixture was added dropwise to distilled water (4.0 mL) under conditions of vigorous stirring (stirring time: 1 h). Subsequently, the mixture was transferred to an ultrafiltration device (MWCO: 10 kDa) and centrifuged at 5000 rpm for 10 min. The system was washed thrice with distilled water, following which the NPs were dispersed in 2 mL of PBS (pH 7.4) to obtain the PRES NPs. To prepare the PTX loaded NPs, 30 μL of the PTX solution (20 mg/mL in DMSO), 150 μL of the PRES solution (20 mg/mL in DMSO), and 180 μL of the DSPE-PEG_3k_ solution (10 mg/mL in DMSO) were mixed, and the mixture was added dropwise to 2 mL of deionized water. The NPs were washed following the protocol described previously. Subsequently, they were dispersed in 2 mL of PBS (pH 7.4) to obtain the PTX@PRES NPs.

The drug loading capacity (DLC) and encapsulation efficiency (DEE) of PTX in PTX@PRES NPs were determined using the HPLC technique. The DLC and DEE were calculated as follows:

(1) $$\mathrm{DLC}\;(\%)=\frac{\text{weight}\;\text{of}\;\text{the}\;\text{drug}\;\text{in}\;\text{NPs}}{\text{weight}\;\text{of}\;\text{NPs}}\times 100\%$$

(2) $$\mathrm{DEE}\;(\%)=\frac{\text{weight}\;\text{of}\;\text{the}\;\text{drug}\;\text{in}\;\text{NPs}}{\text{weight}\;\text{of}\;\text{drug}\;\text{added}\;}\times 100\%$$

### 2.6. Stability of NPs

The changes in the size of the NPs in PBS (pH 7.4; with or without 10% FBS) were detected using the DLS technique to study the stability of the NPs. Freshly prepared solutions of NPs were dispersed in PBS or PBS containing 10% FBS. The final concentration of the solution was maintained at 3 mg/mL. The fabricated NPs were stored at 37 °C under conditions of shaking at 100 rpm. At predetermined intervals (0, 4, 8, 12, 24, 36, and 48 h), 1.0 mL of the solution containing NPs was withdrawn, and the solution was analyzed using the DLS technique.

### 2.7. *In vitro* drug release

The release profiles of RES and PTX from NPs were studied at 37 °C in PBS (pH 7.4) containing 0.5% Tween80 (m/v) using GSH (20 µM or 10 mM) as the release medium. Freshly prepared NPs (10.0 mg) were dispersed (equivalent to 4.3 mg of PRES and 0.7 mg of PTX) in 2.0 mL of Tween80 in the absence of a release medium, and the solution was transferred into a dialysis bag (MWCO: 3.5 kDa). Subsequently, the dialysis bag was immersed into 48 mL of the release medium, and the temperature was maintained at 37 °C under conditions of shaking. At predetermined time intervals, 2 mL of the release medium, present outside the dialysis bag, was withdrawn. The solution was replenished with the same volume of fresh release medium. The amount of RES and PTX present was determined using the HPLC technique.

### 2.8. Cell and animal studies

Human lung cancer cells (A549) and the corresponding PTX resistance cells (A549/PTX) were purchased from KeyGEN Biotechnology Co., Ltd. (Nanjing, China). A549 cells were cultured in F12K containing 10% fetal bovine serum (FBS) and 100 units/mL of streptomycin and penicillin. A549/PTX cells were cultured in RPMI 1640 containing 10% fetal bovine serum (FBS) and 100 units/mL of streptomycin and penicillin. The culture medium was treated with 20 ng/mL of Taxol to maintain the resistance of the A549/PTX cells.

Male BALB/c normal nude mice (4–5 weeks old) were purchased from the Laboratory Animal Center of the USTC. All animal-based experiments were performed in accordance with the guidelines outlined by the National Institutes of Health Guide for the Care and Use of Laboratory animals. The protocol followed for animal-based studies was approved by USTC.

### 2.9. Cellular uptake

The cellular uptake recorded for the NPs was determined using the confocal laser scanning microscopy (CLSM) technique using A549 and A549/PTX cells. Cells (3 × 10^4^) were seeded in round disks and cultured over a period of 24 h. Subsequently, the FBS-free medium containing coumarin 6-loaded NPs were used to replace the medium, and the cells were incubated for another 4 h. Following this, the cells were washed with PBS and then fixed using 4% paraformaldehyde. After staining the nuclei with DPAI, the cells were observed following the CLSM technique.

### 2.10. *In vitro* cytotoxicity

The A549 or A549/PTX cells were seeded in a 96-well plate (density: 5000 cells per well). The cells were incubated over a period of 24 h, following which the medium was replaced by 150 μL of dispersion of NPs and different concentrations of drugs. The cells were incubated for another 48 h. Subsequently, cell viability was analyzed following the CCK-8 assay technique using a Bio-Rad 680 microplate reader at a wavelength of 450 nm. The cell viability was calculated from the data obtained from six parallel wells using the following formula (PBS was used as the negative control):

(3) $$\text{Cell viability }(\%)=\frac{\text{Absorbance}\;\text{value}\;\text{of}\;\text{samples}}{\text{Absorbance}\;\text{value}\;\text{of}\;\text{PBS}}\times 100\%$$

The inhibitory concentration (IC_50_) of each formulation was calculated from the recorded data using Origin 2021b (OriginLab, Northampton, MA, USA).

The resistance index (RI) was calculated following the method presented in literature reports using the following equation:

(4) $$\mathrm{RI}=\frac{\text{IC}50\;\text{of}\;\text{resistance}\;\text{cells}}{\text{IC}50\;\text{value}\;\text{of}\;\text{sensitive}\;\text{cells}}$$

The half-maximal combination index (CI_50_) was calculated to evaluate the synergistic effect of PTX and RES following the method presented in literature reports(Li et al., 2020) using the following equation:

(5) $$R=\frac{D1}{D1x}+\frac{D2}{D2x},$$

where *D*_1_*_x_* and *D*_2_*_x_* represent the IC_50_ value of PTX and RES, respectively, and *D*_1_ and *D*_2_ represent the molar ratio of the two drugs in the combination group at IC_50_. *R* < 1 represents synergy, *R* = 1 represents equivalence, and *R* > 1 represents antagonism.

### 2.11. Pharmacokinetics and biodistribution assay

Sprague Dawley (SD) rat was employed as the animal model to investigate the pharmacokinetic properties of different formulations. In brief, the rats were treated with PTX (5 mg/kg), RES (15 mg/kg), and PTX@PRES NP (equal to 5 mg/kg PTX), respectively, via tail vein injection at a single dose. At pre-set time points, 0.5 mL blood was collected at the orbital vein and immediately centrifuged at 1000 rpm for 3 min to obtain the plasma. Thereafter, 0.2 mL of plasma was mixed with 0.4 mL acetonitrile/water (1:1, v/v) and sonicated for 5 min. After centrifuging at 3500 g for 15 min, the supernatant was collected and detected by HPLC.

Moreover, the biodistribution drug formulations in A549/PTX tumor-bearing mice were also investigated. The murine model was established by subcutaneous injection of 5 × 10^6^ cells to the back region of the BALB/c nude mice. After being implanted for ten days, mice were treated intravenously with PTX or PTX@PRES NPs (equal to 5.0 mg/kg of PTX) *via* the tail vein. After a 24-h treatment, 6 treatment mice were euthanized by rapid cervical dislocating, and the tumors or organs (spleen, kidney, lung, liver, and heat) were collected, weighed, and pulverized. The PTX concentration in each organ was measured by HPLC.

### 2.12. *In vivo* antitumor assay

When the volume of the tumor xenograft reached ∼50 mm^3^, the mice were randomly divided into six groups (*n* = 6). Following this, the mice were treated with PBS, PTX (5 mg/kg), RES (15 mg/kg), PTX + RES (5 mg/kg of PTX and 15 mg/kg of RES), PRES NPs (with an amount equal to the RES dose in PTX@PRES NPs), or PTX@PRES (containing 5 mg/kg PTX), respectively. The samples were injected every two days through the tail vein. Three treatment cycles were conducted over a period of 14 days. The weight of the mouse and the length and width of the tumor was monitored every three days. The tumor volume was calculated as follows:

(6) $$V=(L\times W^{2})/2,$$

where *L* and *W* are the length and width, respectively.

At the experimental endpoint, the mice were sacrificed, and the tumors were harvested and weighed. The tumor inhibitory rate (TIR) was calculated as follows (based on the weight of the excised tumor):

(7) $$\mathrm{TIR}\;(\%)=\frac{1\;-\;\text{tumor}\;\text{weight}\;\text{of}\;\text{test}\;\text{group}}{\text{tumor}\;\text{weight}\;\text{of}\;\text{PBS}\;\text{group}}\times 100\%$$

### 2.13. Statistical analysis

The data were presented as the mean ± standard error by repeating all experiments thrice. Statistical Product and Service Solutions (SPSS; version 17.0) was used for statistical analysis. Significant differences between groups were determined using Student’s *t*-test at *p* < .05, while very significant differences were presented as *p < .05, **p < .01, or ***p < .001.

## 3. Results and discussion

### 3.1. Synthesis and characterization of PRES

**Figure 1.**
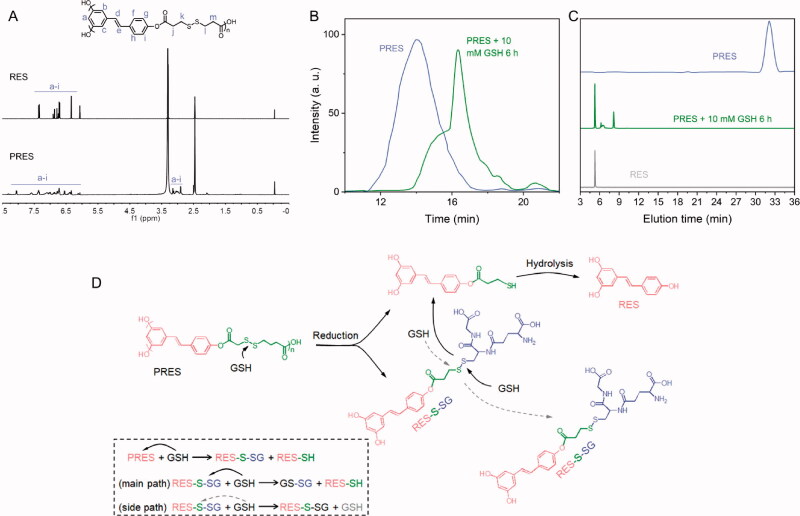
(A) ^1^H NMR spectral profiles recorded for RES and PRES in DMSO-*d6*. (B) GPC spectral profiles recorded for PRES and PRES incubated with 10 mM of GSH (incubation time: 6 h). (C) HPCL spectral profiles recorded for RES, PRES, and PRES treated with 10 mM of GSH (treatment time: 12 h). (D) The GSH-responsive mechanism of PRES.

As mentioned previously, RES, a natural ingredient presents in plants. The use of RES results in almost no side effects. Hence, it was chosen as a model drug and copolymerized with a redox-cleavable disulfide linker to prepare the poly-prodrug (defined as PRES herein). The PRES-based NPs could be easily degraded in the intracellular environment of tumor cells. The synthetic route and structure of PRES are shown in Scheme 1. The samples were characterized using the ^1^H NMR, GPC, MALDI-MS, and HPLC techniques. Analysis of the ^1^H NMR spectrum recorded for PRES (Figure 1(A)) revealed that the hydrogen protons corresponding to DTPA were present in the region of 3.2–3.5 ppm. The broad peaks appearing in the range of 6.0–8.3 ppm could be attributed to the hydrogen protons present in RES. The extended peak shapes suggested the successful polymerization of RES (Zheng et al., 2019). The results obtained using the GPC techniques revealed that the molecular weight (*Mw*) of PRES was 8706 Da (Table S1). It was characterized by a narrow PDI of 1.2 (Figure 1(B) and Table S1). Moreover, the MALDI-MS result showed the average molecular weight of PRES was 8131 Da (Table S1). Since the MALDI-MS is more accurate, the molecular weight of PRES is 8131 Da. Analysis of the chromatogram recorded using the HPLC technique (Figure 1(C)) revealed the presence of a broad peak at 32.1 min (attributable to PRES). The presence of extra peaks was not observed, demonstrating the high purity of PRES. The GPC and HPLC techniques were used to investigate the redox-responsive ability of PRES. When PRES was incubated with 10 mM of GSH for 6 h, the molecular weight of PRES was significantly decreased to ∼300 Da (Figure 1(B)). Under these conditions, the molecular weight was comparable to the molecular weight of free RES. It was observed that following the treatment with 10.0 mM of GSH over 6 h, the retention time for the peak corresponding to PRES in the HPLC spectral profile (Figure 1(C)) decreased significantly. A new peak corresponding to free RES appeared under these conditions., indicating the degradation of PRES into several fragments and free RES. The results suggest that the degradation of PRES is influenced by the GSH-triggered cleavage of the disulfide bond linkages. The GSH-triggered PRES degradation mechanism has been presented in Figure 1(D). The nucleophilic attack by GSH initiated the degradation of PRES and resulted in the formation of RES-SH and RES-S-SG (Zuo et al., 2020). GSH also reacts with RES-S-SG, resulting in the production of oxidized GSH (GSSG) and RES-SH. The nucleophilic -SH group could easily react with the adjacent ester bond, resulting in the rapid hydrolysis of the hydrophilic RES-SH units and the release of RES.

### 3.2. Preparation and characterization of NPs

**Figure 2.**
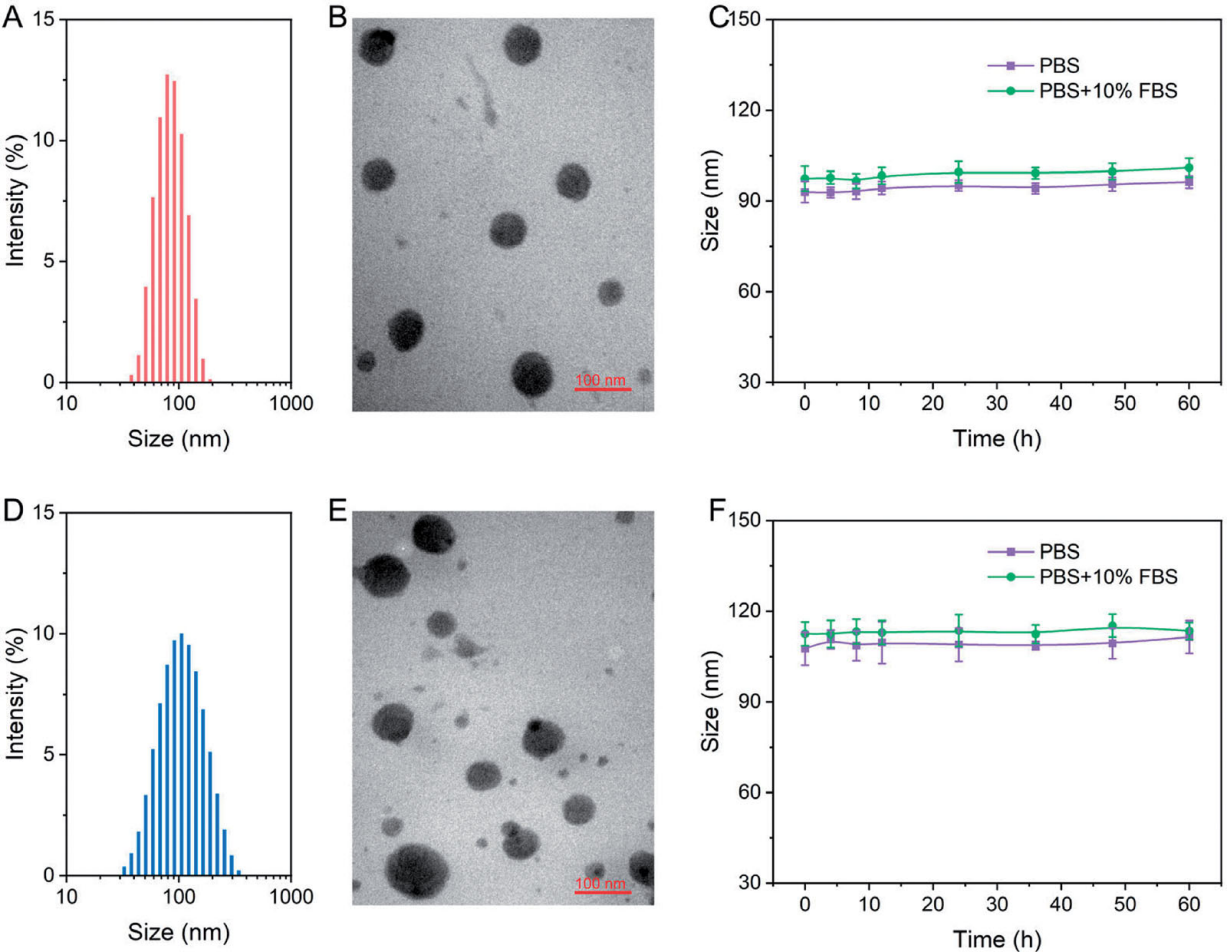
(A–D) Size distribution and TEM images of PRES NPs (A,B) and PTX@PRES NPs (C,D). (E,F) Changes in the sizes of PRES NPs (E) and PTX@PRES NPs (F) in PBS (with or without 10% FBS) at a temperature of 37 °C.

The size and morphology of the NPs dictate their applicability in the field of biological and biomedical fields. These properties also dictate the physiological and pathological conditions required for the efficient treatment of diseases (Zheng et al., 2019). We further investigated whether PRES could be used to construct redox-responsive NPs for on-demand drug release. It is well-known that PEGylated nanomedicines are highly stable and can be used for prolonged blood circulation (Zhao et al., 2016). A biocompatible DSPE-PEG_3k_ was used to achieve good stability and long systemic circulation. A series of NPs containing DSPE-PEG_3k_ (10–50 wt.%; Table S2) was synthesized following a simple nanoprecipitation method. The results revealed that PRES could co-assemble with DSPE-PEG_3k_ (50 wt.%) to form spherical NPs characterized by narrow PDI and appropriate average hydrodynamic size (∼90 nm; Figures 2(A,B), and Table S1). Thus, the optimal DSPE-PEG_3k_ content was found to be 50 wt.%, and 50 wt.% of DSPE-PEG_3k_ was used for further studies. Under these conditions, the drug loading level of RES was calculated in the presence of ∼33.5 wt.% of the PRES NPs. Additionally, the NPs were found to be highly stable in PBS or PBS containing FBS (10%). This was revealed by the fact that the sizes did not change significantly when the NPs were incubated in PBS or PBS containing FBS (10%) for more than 48 h (Figure 1(C)).

We hypothesized that PRES could be used as a drug carrier. PTX was chosen as a model drug to confirm this. PTX is widely used in clinical settings as an anticancer drug, and it has been used for the treatment of breast cancer, lung cancer, and prostate cancer (Sofias et al., 2017). A series of PTX@PRES NPs was prepared under conditions of varying PRES to PTX mass ratios (PTX/PRES = 1:1, 1:3, 1:5, 1:7, or 1:9) to optimize the preparation conditions of PTX@PRES NPs that exhibit good stability and high drug loading ability. The particle size of the prepared NPs ranged from 100 to 190 nm (Table S3). The particle size was small enough to allow excellent tumor accumulation *via* the EPR effect-based passive targeting pathway (Kang et al., 2020). Interestingly, we observed that the NPs were characterized by the maximum DLC (7.2 ± 0.4%) DEE (73.4 ± 4.6%) when the PTX/PRES ratio was 1:5. Hence, the PTX@PRES NPs with a PTX/PRES ratio of 1:5 were used for further studies. Analysis of the TEM images revealed that the PTX-loaded NPs were uniformly distributed and appeared spherical (Figure 2(C)). The PTX@PRESNPs were highly stable, and significant changes in the particle sizes were not observed when they were treated with PBS (with or without 10% FBS) over a period of 48 h (Figures 2(D,E)). This could help reduce the extent of undesired exposure of the parent drug in the blood circulation system and normal cells. Thus, the systemic toxicity of PTX could be reduced.

### 3.3. *In vitro* drug release

**Figure 3.**
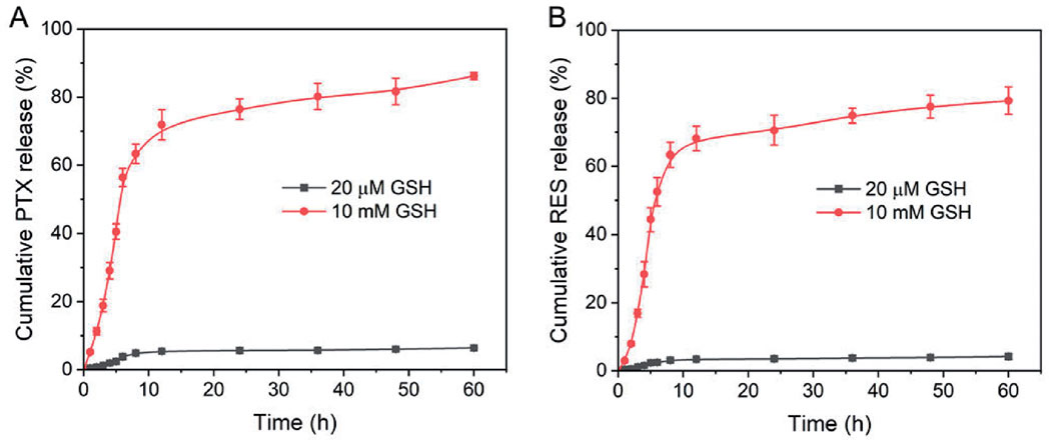
*In vitro* drug release patterns recorded for PTX (A) and RES (B) from the PTX@PRES NPs in the PBS media in the presence of GSH (20 µM or 10 mM).

The concentration of intracellular GSH (2–10 mM) and extracellular GSH (2–20 µM) is different. This redox diversity is an ideal stimulus that can be used to trigger the rapid release of intracellular drugs (Wang et al., 2016). PRES-based NPs could be easily degraded in intracellular environments of tumors. To investigate this, the drug release abilities of the PTX@PRES NPs at various GSH concentrations were determined following the dialysis method. We used PBS (pH 7.4) containing GSH (20 µM or 10 mM) to stimulate blood circulation and tune the intracellular environment. We also used 0.5% Tween80 (m/v) to increase the solubility of the drugs. As presented in Figures 3(A,B), only trace amounts of PTX (∼6%) and RES (∼4%) were released from the PTX@PRES NPs following the incubation (time: 60 h) of the NPs in the blood circulation system containing GSH (20 µM). The results indicated the high colloidal stability of the NPs in the blood circulation system and normal cells. The stable nanostructure of the PTX@PRES NPs could alleviate the reduction and oxidation of the disulfide bonds, resulting in a decrease in the systemic toxicity of PTX. Interestingly, when the GSH concentration was increased to 10 mM (intracellular condition), more than 86 and 79% of PTX and RES, respectively, were released following incubation (time: 60 h). These results suggested the excellent stability of the NPs. The results also revealed that the cargo was not prematurely released during the blood circulation process. The rapid and effective release of drugs could be realized in the cancer cells in the presence of high levels of GSH.

### 3.4. Cellular uptake of NPs

**Figure 4.**
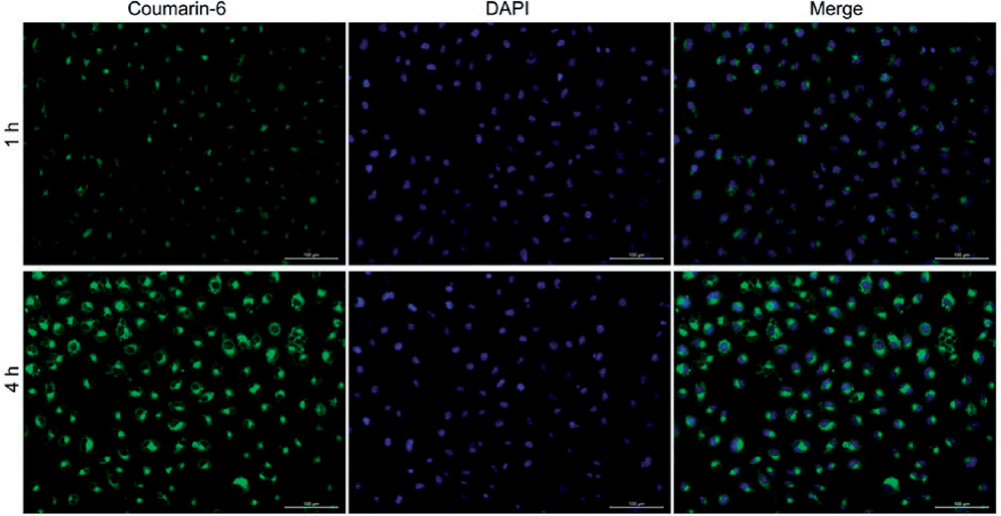
CLSM images of the A549/PTX cells recorded following incubation with coumarin-6-loaded PRES NPs (time: 1 and 4 h).

The NPs should effectively enter cancer cells to achieve efficient intracellular drug delivery. Coumarin-6, instead of PTX, was loaded into PRES NPs as a fluorescence probe to determine cellular uptake. A549/PTX cells were treated with coumarin-6-loaded NPs at 37 °C (treatment time: 2 or 4 h). The image was observed using the CLSM technique. The nuclei were stained with DAPI (blue) for subcellular observation, and the green fluorescence from coumarin-6 was analyzed to visualize the location of the NPs following the internalization of the A549/PTX cells (Figure 4). In the group containing treated NPs, a time-dependent cellular accumulation was observed when the green fluorescent signal recorded at 4 h was significantly stronger than the fluorescent signal recorded at 2 h.

### 3.5. *In vitro* cytotoxicity of NPs

**Figure 5.**
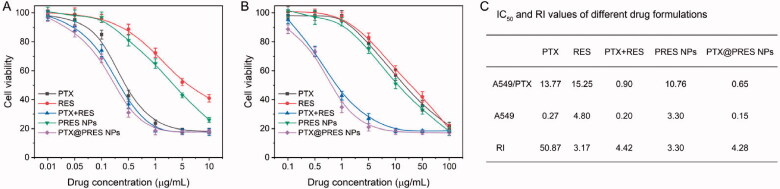
(A,B) Cell viabilities of the A549 (A) and A549/PTX cells treated with free PTX, free RES, free PTX + RES, PRES NPs, and PTX@PRES NPs (*n* = 6). (C) IC_50_ and RI values of each formulation against the A549 and A549/PTX cells.

The *in vitro* cytotoxicity of each formulation was determined following the CCK-8 method and the IC_50_ value was calculated simultaneously (Figures 5(A–C)). The RI value corresponding to PTX (against the A549/PTX and A549 cells) was ∼50.87. The value indicated good resistance capability of the cells. The RI value corresponding to PTX + RES was 4.42. The decrease in the value confirmed that RES could effectively reverse the resistance of cells toward PTX. The RI value recorded for the PTX@PRES NPs was 4.28-fold less than that of free PTX, suggesting that the PTX@PRES NPs could effectively inhibit the growth of cells resistant to various drugs. The PTX@PRES NPs exhibited the maximum cytotoxicity. The lower cytotoxicity recorded for PTX + RES (compared to the cytotoxicity of the PTX@PRES NPs) can be attributed to the poor water solubility of the system. The cytotoxicity of the PRES NPs was higher than the cytotoxicity exhibited by free RES. This could be attributed to the better water solubility of the system. The CI_50_ value was calculated to estimate the synergistic effect, which was 0.32 and 0.21 of PTX + RES and PTX@PRES NPs against A549/PTX cells, respectively. This result demonstrated the synergistic effects of RES and PTX. The combined concentrations of the two drugs were significantly lower than the IC_50_ concentration (when used alone).

### 3.6. Pharmacokinetics and biodistribution

**Figure 6.**
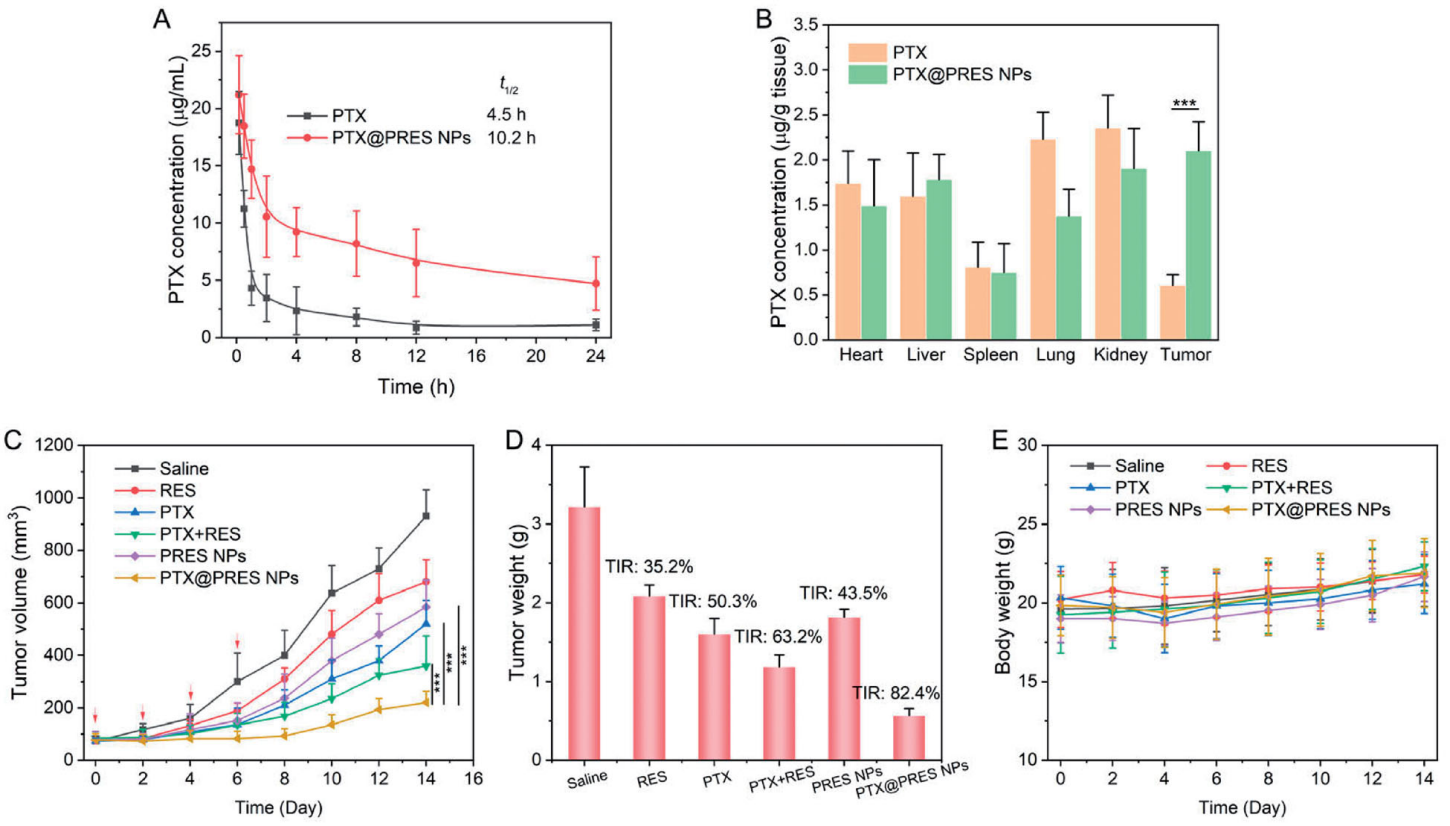
*In vivo* analysis. (A) Pharmacokinetics of free PTX and PTX@PRES NPs in SD rats. (B) Biodistribution of PTX and PTX@PRES NPs in A549/PTX tumor-bearing mice. (C) Changes in the tumor volumes in mice were monitored daily during chemotherapy. (D) The weight of the tumor in each group was recorded on day 14. (E) Changes in the body weights of mice were recorded daily throughout treatment. Data presented as mean ± standard deviation, *n* = 6. ***p < .001.

Long blood circulation enables nanomedicine to accumulate at the tumor tissue through the EPR effect, increasing pharmacological activity (Tee et al., 2019). Thereby, PTX@PRES NPs may remarkably strengthen the blood circulation of RES and PTX. The pharmacokinetics of PTX and PTX@PRES NPs were studied by using SD rats as the animal model. The PTX concentration in plasma *vs.* time curves after intravenous administration of PTX or PTX@PRES NPs is shown in Figure 6(A). The maximum PTX concentration was 17.1 µg/mL and decreased quickly. On the contrary, the maximum PTX concentration in the PTX@PRES NPs group was 21.2 µg/mL, which is 1.2-fold higher than that of free PTX. Moreover, the PTX@PRES NPs prolong the half-life time of free PTX from 4.5 to 10.2 h, demonstrating that the long blood circulation capability of PTX@PRES NPs prodrugs compared to free PTX.

Additionally, the *in vivo* biodistribution study was further performed on A549/PTX tumor-bearing mice to explore the tumor-targeting ability of PTX@PRES NPs. As exhibited in Figure 6(B), after injection for 24 h, the concentrations of PTX in the tumor tissue in the PTX@PRES NPs group were 3.5-fold higher than that of the free PTX group, providing evidence supporting the tumor-targeting ability of PTX@PRES NPs.

### 3.7. *In vivo* antitumor efficacy

The antitumor efficacy was studied in mice bearing A549/PTX tumor to evaluate whether the use of PTX@PRES could result in enhanced therapeutic efficacy. The mice were treated with saline, free PTX, free RES, free PTX + RES, PRES NPs, and PTX@PRES NPs at a PTX dose of 5 mg/kg. The solutions were administered quartic through the tail vein. Tumors in the saline-treated group grew rapidly within 14 days (Figures 6(C,D)). The three ‘free drug’ treatment groups (PTX, RES, and PTX + RES) exhibited moderate antitumor efficacy, and the TIR of REX, PTX, and PTX + RES was 13.2, 15.7, and 16.4%, respectively. The maximum inhibition of cancer cells was observed in the mice treated with PTX@PRES NPs. The TIR of PTX@PRES NPs was 82.3%, which was 1.3-, 1.4-, and 1.5-fold higher than that of the PTX, PTX + RES, and PRES NPs treated group. PRES NPs also exhibited a moderate tumor suppression effect with a TIR of 50.3% (when compared to the effect exhibited by the saline-treated group), suggesting that a high concentration of RES could suppress tumor growth to some extent. Further, the weight of the mice in each group did not change significantly during the period of therapy (Figure 6(E)). This suggested that the drug formulations did not exhibit severe systemic toxicity. Thus, the PTX@PRES NPs can be potentially used to develop an alternative strategy for treating MDR cancer cells.

## 4. Conclusion

In summary, a novel GSH-responsive polymer, PRES, based on RES was successfully prepared. PRES can self-assemble into nanoparticles that can be used in the field of antitumor drug delivery (PTX@PRES NPs). It can also be used to reverse MDR. Results from *in vitro* and *in vivo* studies revealed that the PTX@PRES NPs were stable in blood and could be used to rapidly release drugs under conditions of high GSH concentrations. PRES could also be used to effectively enhance the drug sensitivity of drug-resistant cells. The RES polymer may have potential applications in the field of cancer therapy.

## Supplementary Material

Supplemental Material

## Figures and Tables

**Table 1.** Characteristics of NPs.

NPs	Size (nm)	PDI	DLC (wt.%)		DEE (wt.%)	
			RES	PTX	RES	PTX
PRES	92.9 ± 3.5	0.127 ± 0.005	27.8 ± 4.6	–	72.2 ± 5.4	–
PTX@PRES	107.6 ± 5.4	0.204 ± 0.016	22.8 ± 2.0	7.2 ± 0.4	69.5 ± 5.1	73.4 ± 4.6
