# Supplementary material for: GSH-responsive poly-resveratrol based nanoparticles for effective drug delivery and reversing multidrug resistance
Source: Drug Deliv. 2022 Jan 8;29(1):229–37. doi: 10.1080/10717544.2021.2023700 (PMC8745365; doi:10.1080/10717544.2021.2023700)
Supplement: Supplemental Material [file IDRD_A_2023700_SM9784.docx]

Supporting Information

GSH-responsive poly-resveratrol based nanoparticles for effective drug delivery and reversing multidrug resistance

Liping Yang^#^, Jian He^#^，Zhenchao Tao, Yan Zhou, Jing Yang, Yangyang Zhang，Jing Gao^*^, Liting Qian^*^

Department of Radiotherapy Oncology, the First Affiliated Hospital of USTC, Division of Life Sciences and Medicine, University of Science and Technology of China, Hefei, Anhui, 230031, China

**^#^ These authors have contributed equally to this work**

**Correspondence Authors:**

**1.** Jing Gao.

**Address:** Department of Radiotherapy Oncology, the First Affiliated Hospital of USTC, Division of Life Sciences and Medicine, University of Science and Technology of China, Hefei, Anhui, 230031, China. **Email:** gj20111@163.com

**2.** Liting Qian

**Address:** Department of Radiotherapy Oncology, the First Affiliated Hospital of USTC, Division of Life Sciences and Medicine, University of Science and Technology of China, Hefei, Anhui, 230031, China. **Email:** qlt1964@163.com

**Supporting tables**

| Table S1. GPC and MALDI-MS results of PRES. | | | |
| --- | --- | --- | --- |
|  | *Mw*/Da | *Mn*/Da | PDI |
| GPC | 8706 | 8452 | 1.2047 |
| MALDI-MS | 8131 | - | - |

| **Table S2.** Characterization of PRES NPs with various amount of DSPE-PEG_3k_. | | |
| --- | --- | --- |
| DSPE-PEG_3k_ amount | Size (nm) | PDI |
| 0 | 555.9 ± 26.1 | 0.787 ± 0.063 |
| 10 | 389.5 ± 22.2 | 0.294 ± 0.025 |
| 20 | 264.1 ± 20.3 | 0.132 ± 0.008 |
| 30 | 177.5 ± 11.5 | 0.126 ± 0.006 |
| 40 | 137.2 ± 3.6 | 0.152 ± 0.0145 |
| 50 | 92.9 ± 3.5 | 0.127 ± 0.004 |

| **Table S3.** Characterization of PTX@PRES NPs with various mass ratio of PTX/PRES. | | | | |
| --- | --- | --- | --- | --- |
| Ratio | Size (nm) | PDI | DEE of PTX (%) | DLC of PTX (%) |
| PTX: PRES=1:1 | 185.3 ± 10.0 | 0.399 ± 0.041 | 19.6 ± 2.2 | 5.6 ± 0.7 |
| PTX: PRES=1:3 | 156.6 ± 7.6 | 0.269 ± 0.014 | 40.4 ± 4.8 | 5.9 ± 0.8 |
| PTX: PRES=1:5 | 107.6 ± 5.4 | 0.204 ± 0.016 | 73.4 ± 4.6 | 7.2 ± 0.4 |
| PTX: PRES=1:7 | 121.2 ± 4.9 | 0.245 ± 0.027 | 75.2 ± 5.2 | 4.9 ± 0.5 |
| PTX: PRES=1:9 | 125.7 ± 5.4 | 0.248 ± 0.017 | 74.3 ± 5.3 | 4.3 ± 0.3 |
